# Continuous measurement of radioactivity for a patient with chronic kidney disease during radioactive iodine therapy and hemodialysis: a case report

**DOI:** 10.1007/s13691-025-00756-z

**Published:** 2025-04-09

**Authors:** Takuma Usuzaki, Hiroyasu Kodama, Mariko Miyazaki, Keiichi Jingu

**Affiliations:** 1 Department of Diagnostic Radiology, Tohoku University Hospital, Sendai, Japan; 2 Department of Radiological Technology, Tohoku University Hospital, Sendai, Japan; 3 Department of Nephrology, Tohoku University Hospital, Sendai, Japan; 4 Department of Radiation Oncology, Tohoku University Hospital, 1-1 Seiryo-Machi, Aoba-Ku, Sendai, Miyagi 980-8574 Japan

**Keywords:** Hemodialysis, I-131 therapy, Thyroid cancer

## Abstract

The half-life of radioactive iodine (RAI) is prolonged in patients with chronic kidney disease (CKD) because RAI is mainly excreted by the kidneys. There is little information on the RAI half-life in patients with dialysis-dependent CKD (CKDG5d). Estimating the RAI half-life in a patient’s body provides important information for treatment planning. In this paper, we report a 68-year-old woman of CKDG5d who underwent postsurgical RAI therapy for papillary adenocarcinoma of the thyroid. We administered 15 mCi (0.56 GBq) RAI (^131^I) and continuously measured the dose equivalent rate. The results were summarized into hourly values of dose equivalent rate. Based on the measurements, we estimated the RAI half-life in the patient’s body using a semi-log plot and linear regression analysis. In addition, we calculated the integrated doses for caregivers and the public using coefficients of 0.5 and 0.25, respectively. The half-life in the patient’s body was 7.2 days (95% confidence interval, 4.8–14.4). The integrated doses for caregivers and the public were 0.23 mSv and 0.11 mSv, respectively. RAI therapy for a CKDG5d patient should be planned on the basis of the biological dynamics of ^131^I. Accumulation of more cases should lead to the establishment of a treatment strategy for patients undergoing RAI therapy and hemodialysis.

## Introduction

Thyroid cancer is the most common endocrine malignancy, and its incidence is approximately 8.0 and 22.8 per 100,000 per year for men and women, respectively [1, 2]. Administration of radioactive iodine (RAI) ^131^I is a standard treatment for differentiated thyroid cancer [3]. The RAI therapy plan and dose recommendation change depending on the risk and patient’s conditions: thyroid remnant ablation, adjuvant radiation, and treatment of metastasis or recurrence [4, 5]. Previous studies have shown that RAI therapy resulted in significant improvements in overall mortality and disease-specific mortality as well as disease-free survival in high-risk patients [6, 7].

Although recommendations for RAI therapy have been established for patients with normal renal functions, how to plan postsurgical RAI therapy for patients with chronic kidney disease (CKD) remains controversial [4, 8–19]. In Japan, the five-year survival rate of patients with dialysis-dependent CKD (CKDG5d) patients is approximately 60%, which is higher than that in the United States (39%) and that in Europe (41%) [20, 21]. Treatment for CKDG5d patients with cancer in Japan is aimed at prolonging survival time if systemic conditions allow and the advantages of treatment outweigh the disadvantages. Based on this situation, the number of CKDG5d patients who need postsurgical RAI therapy may increase in the future with the aging of the population in Japan. RAI therapy is not performed for CKDG5d patients in some facilities due to difficulties in radiation exposure management [22]. The half-life of RAI is prolonged in patients with CKD because RAI is mainly excreted by the kidneys (90%), and the prolongation of the RAI half-life increases radiation exposure for the patients themselves, caregivers and the public [23]. In addition, clearance of RAI by hemodialysis can be another problem for effective treatment for CKDG5d patients. In previous studies, attempts were made to estimate [17, 18] and measure [18] the activity of RAI after administration of RAI in CKDG5d patients. However, more detailed changes in the dose equivalent rate during RAI therapy with hemodialysis need to be investigated to determine treatment planning. In this paper, we report a CKDG5d patient who underwent postsurgical RAI therapy and hemodialysis with the dose equivalent rate of RAI measured every hour.

## Literature review

Patients who underwent postsurgical RAI therapy for thyroid cancer and hemodialysis have been reported [4, 8–19]. These studies are summarized in Table 1. As far as we researched, the first case was reported by Howard et al. in 1981 [8]. After that report, 24 cases were reported. The dose varied from 25 mCi (0.93 GBq) to 250 mCi (9.3 GBq) depending on the conditions of each case. Regarding the number of administrations for one patient, divided or repeated administration was performed in five cases [9, 12, 18]. Morish et al. treated one patient four times with increases in the dose [9]. Daumerie et al. administered 25 mCi (0.93 GBq) two times with a six-month interval [12]. The timing of hemodialysis also varied depending on the treatment strategy. Daily hemodialysis was planned in some studies [4, 13, 16]. The largest case series was reported by Vermandel M. et al. They reported 6 cases with their TSH levels and changes in radioactivity measured by a γ-counter during treatment [18]. The dose ranged from 50 mCi (1.85 GBq) to 100 mCi (3.75 GBq) depending on the risk of recurrence. Dialysis was scheduled for 40 h and 90 h after administration of RAI. They measured TSH levels eight times for each patient. A notable point in their case series is that they estimated the bone marrow absorbed dose using medical internal radiation dosimetry (MIRD) formalism [24]. The total absorbed dose in bone marrow ranged from 0.431 to 2.23 Gy. A unique attempt was performed by Gallegos-Villalobos et al. [16]. In addition to the dose equivalent rate measured at 1 m from the patient, they reported the accumulated dose in a nurse.

**Table 1:** Literature summary on radioactive iodine therapy for thyroid cancer with hemodialysis (n = 24)

Article	Age (year)	Gender	Diagnosis	Dose (mCi)	Hemodialysis planning
Howard N. and Glasser M [8]	34	F	PTC	80	48 h
Morrish D. W. et al. [9]	36	M	PTC	50, 120, 150, 250 (repeated in 4 years)	24–48 h
Culpepper R. M. et al. [10]	56	F	FTC	129	24, 43, 66 h
Mello A. M. et al. [11]	42	F	PTC	100	41, 98 h
Daumerie C. et al. [12]	42	F	PTC	25 (2 sessions with 6 months interval)	2, 5, 7 d
	62	F	PTC	25 (2 sessions with 6 months interval)	2, 5, 7 d
	27	–	PTC	25 (2 sessions with 6 months interval)	2, 5, 7 d
Jimenez R. G. et al. [13]	42	M	PTC	75	Daily for 5 d
	51	M	PTC	87	Daily for 5 d
	34	M	PTC	120	Daily for 5 d
Magne N. et al. [14]	43	M	PTC	50	1, 3, 6 d
Sinsakul M. and Ali A [15]	43	F	PTC	100	20 h
	56	M	PTC	157	24 h
Holst J. P. et al. [4]	40	F	PTC	90	2, 3, 4 d
Gallegos-Villalobos A. et al. [16]	51	M	PTC	100	1, 2 d
	52	F	PTC	100	1, 2 d
Bhat M. et al. [17]	49	F	PTC	50	15, 27, 43 h
Vermandel M. et al. [18]	67	F	PTC	60	42, 90 h
	47	M	PTC	77, 82	42, 90 h
	62	M	PTC	61	42, 90 h
	63	M	PTC	50	42, 90 h
	29	F	PTC	60	42, 90 h
	71	M	VC	101	42, 90 h
Kumar M. et al. [19]	35	M	PTC	50	48, 72, 96 h

Gender: F and M represent female and male, respectively

1 mCi = 37 MBq

*PCT* papillary thyroid carcinoma, *VC* vesicular carcinoma, *FTC* follicular thyroid carcinoma

## Case report

A 68-year-old woman was referred to our hospital from Hospital A for postsurgical RAI therapy for papillary adenocarcinoma of the thyroid. About four months before visiting our hospital, she underwent total thyroidectomy and D2 lymph node dissection at Hospital A. A preoperative CT scan showed that there was no distal metastasis. The tumor had invaded into tracheal cartilage, cricoid cartilage, and esophageal serosa. A part of the tumor that had invaded into tracheal cartilage and cricoid cartilage may have remained. Pathological assessment revealed that the tumor had infiltrated into surrounding connective tissue, anterior cervical muscles, and the recurrent laryngeal nerve. Lymph node metastasis with extra-nodal extension was observed in 8 out of 18 lymph nodes. The final diagnosis was papillary adenocarcinoma (pT4aN1b-2, Stage III). She was a candidate for adjuvant RAI therapy. The patient had CKDG5d associated with diabetes mellitus and IgA nephropathy. Hemodialysis had been performed every Monday, Wednesday, and Friday at Clinic B for 3 years. Her other medical history included hypertension, cataracts, coronary arterial disease, cerebral aneurysm, femoral fracture, and colon cancer. The patient’s medications were levothyroxine sodium hydrate, linagliptin, aspirin, lansoprazole, atorvastatin calcium hydrate, alfacalcidol, bisoprolol fumarate, shakuyakukanzoto, lanthanum carbonate hydrate, acetaminophen, rebamipide, and nicorandil. Before performing RAI therapy, we confirmed the absence of contraindications to the treatment.

### Treatment and hemodialysis schedule

One week before hospitalization, the patient had taken a low iodine diet. On the day of hospitalization, the patient was admitted to our hospital after undergoing hemodialysis at Clinic B. We followed the standard thyrotropin alfa regimen for patients with preserved renal function because the patient would undergo hemodialysis on the fourth hospital day. In a previous study, a single injection of thyrotropin alfa 48 h before ^131^I administration was used to avoid an excessive level of thyroid-stimulating hormone (TSH) [18]. We dissolved 0.9 mg of thyrotropin alfa in 1.2 ml of an injection solvent and injected it into the muscle at 48 h (on the second hospital day) and 24 h (on the third hospital day) before ^131^I administration. On the fourth hospital day, after hemodialysis, ^131^I (15 mCi, 0.56 GBq) was administered. On the seventh hospital day, hemodialysis was performed at our hospital. On the eighth hospital day, thyroid scintigraphy was performed. The patient was discharged on the ninth hospital day.

### Hemodialysis

Four hours of hemodialysis was performed through an arteriovenous fistula (AVF) using a VPS-15HA with a Qb of 220 ml/min and a Qd of 500 ml/min. The dialysis staff and all visitors were required to wear protective overgarments and lead shielding; glass badges were worn to monitor radiation exposure. After hemodialysis, all the devices were collected and kept in a shielded room until the radiation dose rate dropped.

### Dose rate measurement

During hospitalization, two types of radiation measurements were performed. First, the dose equivalent rate was measured every hour using a device (device 1). The device 1 was placed about 90 cm apart from the patient. Second, the dose equivalent rate was measured using a γ survey meter (device 2). The dose equivalent rate was measured about 1 m from the patient by a medical doctor with 4 years of experience. The doctor checked the dry battery in every measurement. The first measurement was made immediately after administration of ^131^I. After the administration, the measurements were performed 11 times during hospitalization.

### Estimation of half-life

We estimated the half-life of ^131^I in the patient’s body from administration of ^131^I to hemodialysis as follows. In the estimation, we standardized the values measured by device 1 to values measured at 1 m apart from the patient.

i) We assumed that the dose equivalent rate changes according to
  $$D\left(t\right)={D}_{0}\text{exp}\left(-\frac{\text{ln}2}{{T}_{1/2}}t\right),$$

where ${D}_{0}$ and ${T}_{1/2}$ represent dose equivalent rate at $t=0$ and half-life, respectively.

ii) A semi-log plot was applied to assumption i),and the slope (α) was estimated using the least square method. The estimated slope was statistically tested according to linear regression analysis.
  $$lnD\left(t\right)={lnD}_{0}-\frac{\text{ln}2}{{T}_{1/2}}t,$$

iii) We obtained the half-life ${T}_{1/2}$ as ${T}_{1/2}=-\frac{\text{ln}2}{\alpha }$.

### Estimation of integrated dose for caregivers and the public

We estimated the integrated dose for caregivers and the public based on the guide for the appropriate use of ^131^I in RAI therapy [25]. In this guide, it is recommended that the integrated dose for a caregiver be under 5 mSv according to the ICRP Publication 73 [26]. As an exception, the integrated dose for a child should be under 1 mSv. Similarly, the guide recommends that the upper limit of the dose should be 1 mSv per year, according to the ICRP Publication 103 [27]. Based on these values, the guide sets the dose conversion coefficients for caregivers and the public as 0.5 and 0.25, respectively. In Japan, there is another guide for ablation therapy using 1,110 MBq of ^131^I: a guide for ablation therapy using 131I (1,110 MBq) at an outpatient clinic [28]. This guide sets the dose conversion coefficients for caregivers and the public as 0.25 and 0.25, respectively. The difference between the two guides is explained by the fact that a patient undergoes ablation therapy using ^131^I (1,110 MBq) at an outpatient clinic on the condition that the patient independently lives under some restrictions to avoid radiation exposure to caregivers and the public. Since it is controversial whether the value of 0.5 or 0.25 should be used for a patient undergoing RAI therapy and hemodialysis, we calculated caregivers’ exposure using the values of 0.25 and 0.5. Both guides set the coefficient for internal exposure due to inhaling as 1.045, and we used this value. The calculation was performed from the administration of ^131^I to hemodialysis and from the administration of ^131^I to discharge.

## Results

**Fig. 1:**
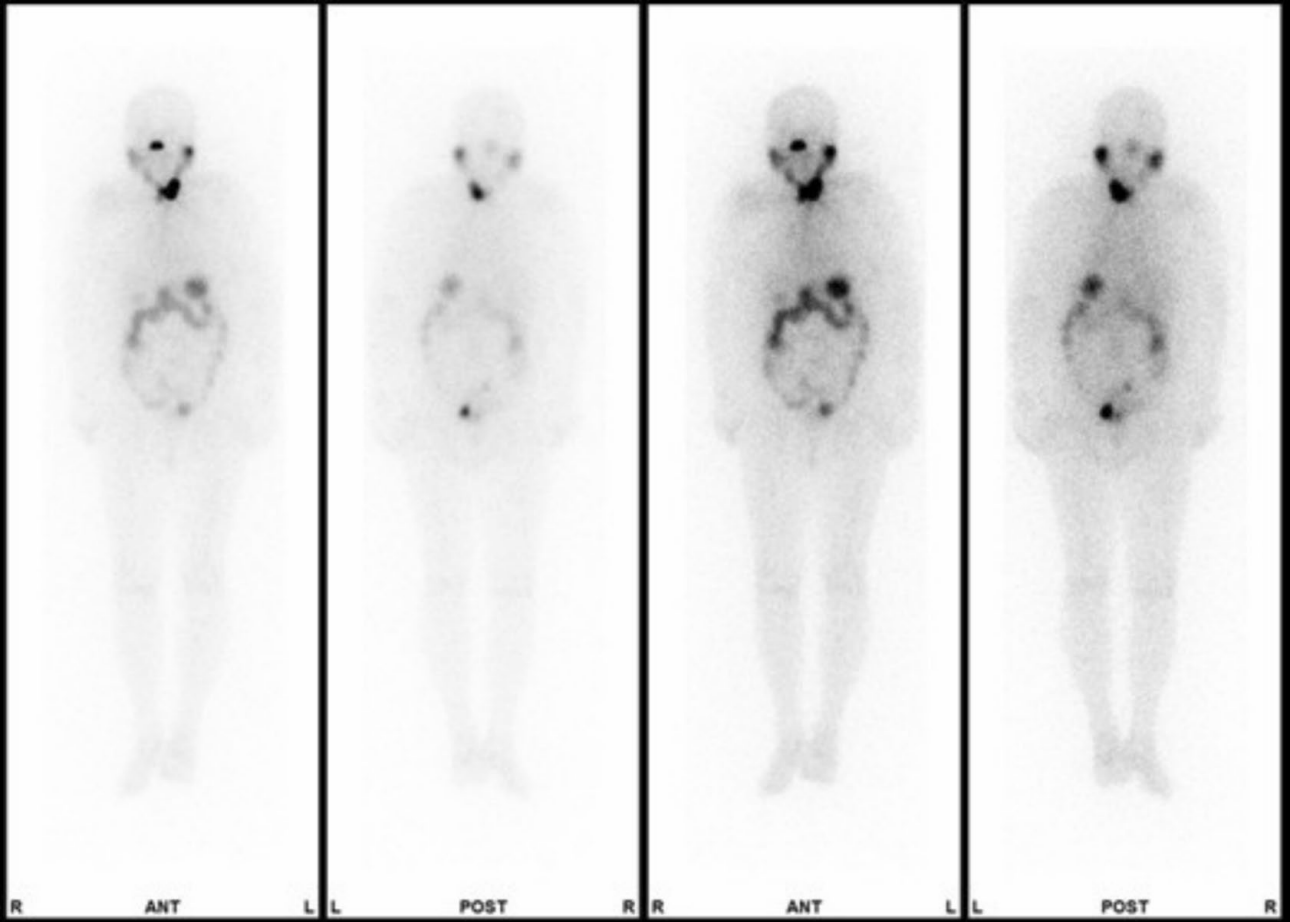
Results of thyroid scintigraphy performed on the eighth hospital day. Accumulation of ^131^I was observed in the remaining thyroid tissue. Physiological accumulation on salivary glands, nasal mucosa, stomach, and other parts of the digestive tract was observed

During hospitalization, the patient had no complications associated with thyrotropin alfa, RAI therapy, and hemodialysis. Laboratory tests performed on the fourth hospitalization day before ^131^I administration showed that free T4 was 1.43 ng/dL (reference range: 0.90–1.70), free T3 was 1.89 ng/dL (2.30–4.00), human TSH was 372 μIU/mL (0.50–50.0), thyroglobulin was 3.3 ng/mL (3.7–35.1), and thyroglobulin antibody was 19.5 IU/mL (< 19.3). Blood urea nitrogen (BUN) and creatinine (Cre) were 35 mg/dL and 8.04 mg/dL, respectively. Her estimated glomerular filtration rate (eGFR) was calculated at 4.0 mL/min/1.73m^2^. Figure 1 shows the results of thyroid scintigraphy on the eighth hospital day. As shown in the thyroid scintigraphy image, ^131^I accumulated on the remaining thyroid tissue. Physiological accumulation on salivary glands, nasal mucosa, stomach, and other parts of the digestive tract was observed. Accumulation in the bladder was not observed.

### Change in the dose equivalent rate measured by device 1

**Fig. 2:**
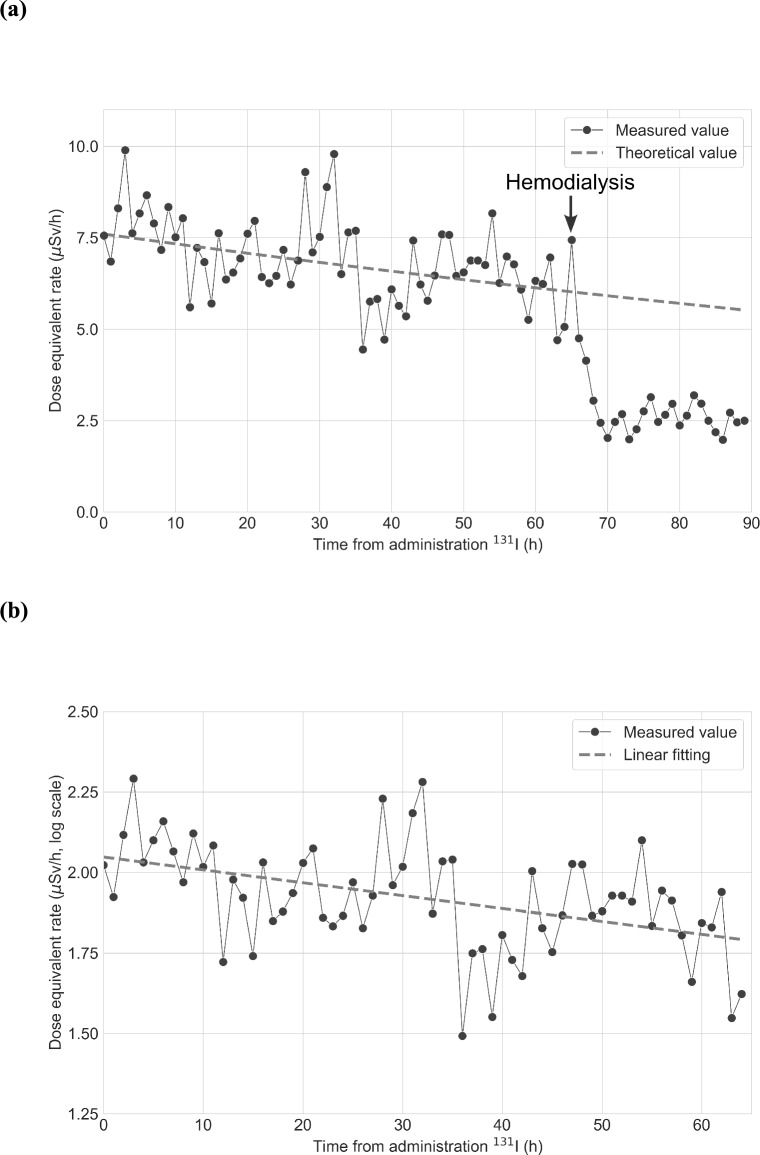
Change in the dose equivalent rate measured by device 1. **a** The dose equivalent rate after administration of ^131^I (15 mCi, 0.56 GBq) is shown. The gray broken line represents the theoretical value with physical half-life. The vertical and horizontal axes show the dose equivalent rate (μSv/h) and time from administration of ^131^I (h), respectively. **b** The dose equivalent rate from administration of ^131^I to hemodialysis using a semi-log plot is shown. The gray broken line represents the linear fitting. The vertical and horizontal axes show the logarithm of the dose equivalent rate (μSv/h) and time from administration of ^131^I (h)

On the second hospitalization day, the mean dose equivalent rate was 0.0049 µSv/h (95% CI 0.036–0.063). We used this value as the background dose equivalent rate. After administration of ^131^I, the dose equivalent rate increased to 7.6 µSv/h. After administration of ^131^I, the dose equivalent rate was continuously measured by device 1, and we obtained the dose equivalent rate every hour. Figure 2a and b show the change in the dose equivalent rate and its semi-log plot, respectively. The slope in the semi-log plot in Fig. 2b was estimated to be –0.0040 /h (95% CI **– **0.0060 to **– **0.0020; p-value < 0.0001). The half-life in the patient’s body was estimated to be 7.2 days (95% CI 4.8–14.4). The integrated dose administration of ^131^I to hemodialysis was 0.46 mSv. The integrated doses for caregivers using the dose conversion coefficients of 0.5 and 0.25 were 0.46 × 0.5 × 1.045 = 0.24 mSv and 0.46 × 0.25 × 1.045 = 0.12 mSv, respectively. The integrated dose for the public was 0.46 × 0.25 × 1.045 = 0.12 mSv. The integrated dose administration of ^131^I to discharge was 0.52 mSv. The integrated doses for caregivers using the dose conversion coefficients of 0.5 and 0.25 were 0.52 × 0.5 × 1.045 = 0.27 mSv and 0.52 × 0.25 × 1.045 = 0.14 mSv, respectively. The integrated dose for the public was 0.52 × 0.25 × 1.045 = 0.14 mSv.

### Change in the dose equivalent rate measured by device 2

**Fig. 3:**
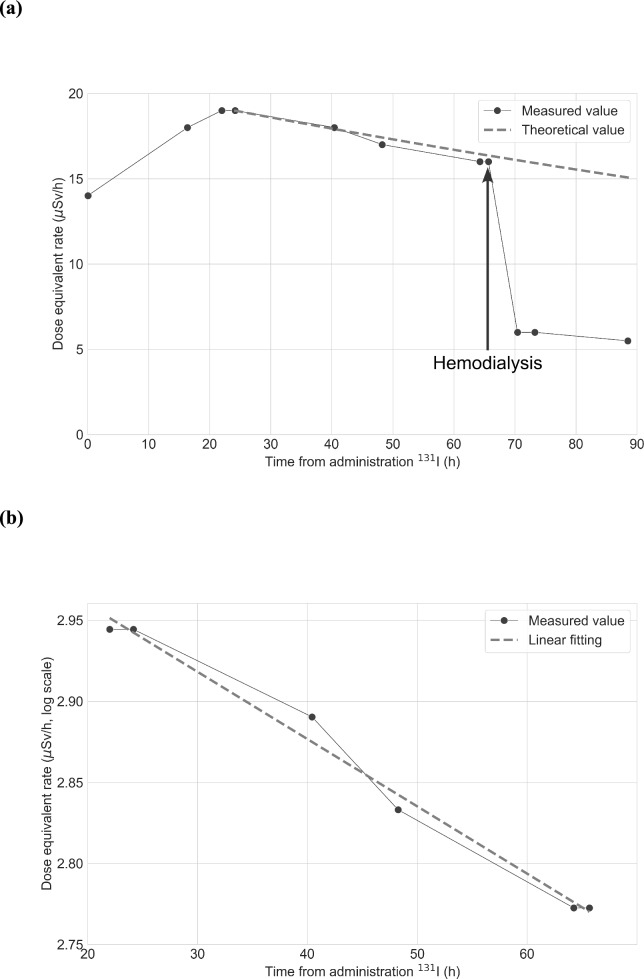
Change in the dose equivalent rate measured by device 2. **a** The dose equivalent rate after administration of ^131^I (15 mCi, 0.56 GBq) is shown. We measured the dose equivalent rate at 1 m from the patient every time. The gray broken line represents the theoretical value with the physical half-life. The vertical and horizontal axes show the dose equivalent rate (μSv/h) and time from administration of ^131^I (h), respectively. **b** The dose equivalent rate from administration of ^131^I to hemodialysis using a semi-log plot is shown. The gray broken line represents the linear fitting. The vertical and horizontal axes show the logarithm of the dose equivalent rate (μSv/h) and time from administration of ^131^I (h)

We measured the dose equivalent rate 11 times using device 2 as shown in Table 2. Figure 3a and b show the change in the dose equivalent rate and its semi-log plot, respectively. The slope was estimated to be **–**0.0041 /h (95% CI **–**0.0048 to **–**0.0035; p-value < 0.0001). The half-life in the patient’s body was estimated to be 7.0 days (95% CI 6.0–8.3). The integrated dose administration of ^131^I to hemodialysis was 1.1 mSv. The integrated doses for caregivers using the dose conversion coefficients of 0.5 and 0.25 were 1.1 × 0.5 × 1.045 = 0.57 mSv and 1.1 × 0.25 × 1.045 = 0.29 mSv, respectively. The integrated dose for the public was 1.1 × 0.25 × 1.045 = 0.29 mSv. The integrated dose administration of ^131^I to discharge was 1.3 mSv. The integrated doses for caregivers using the dose conversion coefficients of 0.5 and 0.25 were 1.3 × 0.5 × 1.045 = 0.68 mSv and 1.3 × 0.25 × 1.045 = 0.34 mSv, respectively. The integrated dose for the public was 1.3 × 0.25 × 1.045 = 0.34 mSv.

**Table 2:** Measurement using device 2

Time from administration of ^131^I (h)	Dose equivalent rate(μSv/h)
0	14
16	18
22	19
24	19
40	18
48	17
64	16
66	16
70	6.0
73	6.0
89	5.5

15 mCi (0.56 GBq) was administered

## Discussion

A patient with CKDG5d who underwent postsurgical RAI therapy (15 mCi, 0.56 GBq) was described in this case report. To our knowledge, this is the first continuous measurement for the dose equivalent rate and estimation of the ^131^I half-life in the patient’s body. The ^131^I half-life was estimated by using device 1 to be 7.2 days (95% CI 4.8–14.4) and it was estimated by using device 2 to be 7.0 days (95% CI 6.0–8.3). Half-life estimations by the two devices were similar. The ^131^I half-life in the patient’s body was shorter than the physical half-life of ^131^I, 8.02 days. This can be explained by the excretion pathways of ^131^I. The main path of ^131^I excretion after administration is urine, and about 70% and about 80 to 90% of ^131^I is excreted in the first 24 h and 48 h, respectively [29, 30]. The other excretion paths are sweat, feces, and saliva [31, 32]. For patients with CKD, the main path fails to function, and the ^131^I half-life in the body is prolonged, especially, in patients with CKDG5d. The 95% CI using device 1 was wider than that using the γ survey meter. As a reason, the distance between device 1 and the patient caused this difference in the 95% CI. The distance varied depending on the patient’s position and posture associated with actions, such as eating, sleeping, using the restroom, and bathing, because we fixed device 1 on the wall. On the other hand, the patient had the same posture and distance from device 2 in every measurement. Hanging device 1 around the patient’s neck may be a solution.

As summarized in the literature review, various treatment plans have been performed [4, 8–19]. In our case, we administered 15 mCi (0.56 GBq), and the dialysis interval was 68 h after ^131^I administration. Clearance of ^131^I is 4–5 times higher through hemodialysis than through renal excretion, and the half-life of ^131^I during hemodialysis was estimated to be 2–4 h [10, 12]. The dose of ^131^I ranged from 25 to 250 mCi depending on the patient’s condition in previous studies. We determined the maximum dose for our case under the two conditions: (1) she had to leave the isolation ward and undergo hemodialysis with other common patients because the hemodialysis machine cannot be placed in the isolation ward, and (2) family members living in the same home must care for the patient and cannot avoid exposure to radiation. We administered 15 mCi (0.56 GBq) for our case to satisfy the two conditions. The appropriate dose might be 50 mCi, which is a half dose for patients with normal renal function according to the previous studies for adjuvant therapy [9, 14, 17, 19]. The effectiveness of the treatment may be limited; however, we believe that the treatment was the best under the patient’s and our facility’s situation. Effective treatment should be performed, but it is also important to reduce radioactivity in order to avoid unnecessary exposure for the patient and others. How to plan hemodialysis during RAI therapy is still controversial. The estimated half-life in the present study may provide important information for planning hemodialysis.

In the present case, we estimated the integrated doses for caregivers and the public based on a Japanese guide. These values were lower than the reference values for adults. On the other hand, when more than 30 mCi (1.11 GBq) of ^131^I is administered at an outpatient clinic without hospitalization, the integrated dose can be higher than the reference value for a child (1 mSv). When a patient is administered more than 30 mCi (1.11 GBq) at an outpatient clinic, the patient should not be with a child until hemodialysis. This proposal regarding contact with children is consistent with the Japanese guide: when the s with a pregnant woman or child, ablation therapy using RAI should not be performed at an outpatient clinic. In Japan, the main reason why not performing RAI therapy at a hospital is that a patient needs hemodialysis [22]. Although a physician faces difficulty in performing RAI therapy for a patient with CKD, RAI therapy can be performed by understanding the radiation exposure for caregivers and the public during treatment as well as for the patient.

Moreover, the difficulty in performing dialysis therapy in times of disasters has been recognized for a long time in Japan. [33]. We experienced the Great East Japan Earthquake Disaster 2011 and the Fukushima Daiichi nuclear power plant accident. Although no dialysis patients were exposed to radiation from the accident, we should prepare to perform dialysis after radioactive contamination [34]. The information obtained from the present study should be useful not only in normal circumstances but also when there are emergencies involving radiation such as radiological accidents or terrorism that are included in chemical, biological, radiological, nuclear, and explosive (CBRNE) disasters [33].

## Conclusion

We reported a 68-year-old woman with CKDG5d who underwent postsurgical RAI therapy for papillary adenocarcinoma with scheduled hemodialysis. We continuously measured the dose and the dose equivalent rate, and estimated the biological half-life of ^131^I in the CKDG5d patient. We should plan treatment based on the biological dynamics of ^131^I in each patient. Accumulation of more cases and long-term follow-up data are needed to establish a treatment strategy for such patients.

## Data Availability

The authors declare that all the data in this article are available within the article.
